# Mapping multispectral Digital Images using a Cloud Computing software: applications from UAV images

**DOI:** 10.1016/j.heliyon.2019.e01277

**Published:** 2019-03-01

**Authors:** Jose Ramon Saura, Ana Reyes-Menendez, Pedro Palos-Sanchez

**Affiliations:** a Department of Business Economics, Rey Juan Carlos University, Paseo Artilleros s/n, 28032, Madrid, Spain; b Department of Business Organization, Marketing and Market Research, International University of La Rioja, Av. de la Paz, 137, 26006 Logroño, Spain

**Keywords:** Computer science, Environmental science, Geoscience

## Abstract

Due to technology development related to agricultural production, aircrafts such as the Unmanned Aerial Vehicle (UAV) and technologies such as Multispectral photogrammetry and Remote Sensing, have great potential in supporting some of the pressing problems faced by agricultural production in terms of analysis and testing of variables. This paper reports an experience related to the analysis of a vineyard with multispectral photogrammetry technology and UAVs and it demonstrates its great potential to analyze the Normalized Difference Vegetation Index (NDVI), the Near-Infrared Spectroscopy (NIRS) and the Digital Elevation Model (DEM) applied in the agriculture framework to collect information on the vegetative state of the crop, soil and plant moisture, and biomass density maps of. In addition, the collected information is analyzed with the PIX4D Cloud Computing technology software and its advantages over software that work with other data processing are highlighted. This research shows, therefore, the possibility that efficient plantations can be developed with the use of multispectral photogrammetry and the analysis of digital images from this process.

## 1 Introduction

Over the last decade new technologies have changed the way industries and businesses develop and improve their products and services [1]. As a consequence of technology advances, drones, also known as Unmanned Aerial Vehicles (UAVs), are playing a key role in addressing problems faced by agricultural production [2].

UAV is a tool for improving agricultural cultivation, increasing yield performance and optimizing resources and hence, maximizing the benefits while optimizing the costs [3]. In this way, we can highlight that different wineries around the world use UAVs to improve operations which aim at optimizing indicators related to irrigation, production, ripening or pests, to name a few.

The development of theses researches in the agricultural sector has led to the evolution and use of multispectral photogrammetry technology, which identifies the different stages of production such as fruit set, version or ripening by analyzing the generated images [1]. Multispectral photogrammetry captures information using on-board multispectral sensors. This technology carries out an exhaustive analysis of the surfaces it analyses, considering factors such as variability, water status, development and quality of the plants, vegetative state, soil moisture and produced biomass [5].

The development of technologies such as multispectral photogrammetry accompanied by remote drone technology helps to identify and increase the great potential of terrains in terms of crop homogeneity or adjustment for fertilization. The Multispectral UAV photogrammetry technique [6] provides very useful information that facilitates the decision on the optimal time to harvest, the treatments to be applied in each area of the crop or the need for irrigation based on energy efficiency models. In addition, multispectral photogrammetry can identify emerging pest, vegetation issues, as well as residual areas under control. With the use of multispectral photogrammetry and UAVs we can find not only improvements in the agriculture but also in industries related to security, professional emergencies or business environment. One of these improvements is the implementation of unmanned vehicles in real interventions or their integration in industrial processes that can minimize personal risks, increase response capabilities or lower costs, among other benefits [6, 7].

In this way, it is interesting to notice the role of UAVs in the agricultural sector with respect to different technologies that can be utilized with multispectral photogrammetry [1, 5, 6], as shown in Table 1.

**Table 1:** Comparison of UAV with other manned airborne and satellite platforms.

	Spatial Resolution	Field of View	Usability	Payload Mass	Cost for Adat Acquisition
UAV	0.5–10cm	50–500m	Very good/easy	Can be limited	Very low
Helicopter	5–50 cm	0.2–2km	Pilot mandatory	Almost unlimited	medium
Airborne	0.1–2m	0.5–5km	Pilot mandatory	unlimited	high
Satellite	1–25m	10–50km	-	-	Very high, particularly for high-res stereo imagery

This study aims to analyze a vineyard with the multispectral photogrammetry and UAVs technology to demonstrate its usefulness regarding the vegetative state of the crop, the analysis of soil and plant moisture, as well as the volume maps of produced biomass through the analysis of the Normalized Difference Vegetation Index (NDVI), Near-Infrared Spectroscopy (NIRS) and Digital Elevation Model (DEM) [8].

It highlights the possibility that efficient plantations can be developed using this technology. In addition, it is organized as follows: Section 1 describes research motivations and objectives. Section 2 shows the view of the scientific community on the present topic through previous works. The methods including the experimental area are described in Section 3 followed by the outline of the data collection process and the operator used to perform the study. Finally, Section 4 focuses on the used methodology and the exploratory analysis of the vegetation indices as a development of the multispectral images process [1, 9].

Likewise, multispectral photogrammetry with UAVs is a newly adopted technique in the primary sector. It consists of an aerial inspection process of aeroponic cultivation which provides information on crop health, coloration, humidity level and other values. The information is collected from the plant at different wavelengths [1, 10].

This research collects critical considerations as well as key indicators for the accuracy and the use of a multispectral UAV photogrammetry to improve vineyards [11]. With photogrammetric methods and high-resolution digital images, this experiment evaluates the potential development of the vineyard maps as the research object. In particular, it focuses on:

• The photogrammetric planning and processing of multispectral images captures with a camera mounted on an UAV;
• The creation and analysis of high-resolution images over the vineyard as the research object;
• The generation and assessment of high-resolution vineyard maps
• The analysis of VI in order to determine the vegetative stage of crop
• The analysis of soil and plant moisture by NIR
• The analysis of biomass maps extracted from the vineyard

These objectives have been defined considering the approach methodology used to understand and clearly identify the most efficient way to deliver geo-referenced information regarding the analysis of medium-sized vineyard plantations [1, 12]. In addition, this study contributes to improve knowledge in this field and its results, can be used by professionals who want to improve their self-consumption or those who want to produce optimal vineyards based on the techniques presented in this study.

## 2 Background

UAV images and other photogrammetric products enable us to obtain in a fast and easy way strategies to measure the accuracy on different characteristics and to manage, among other options, information and data related to the agricultural surface or terrain. For example, some researches focus on the development of experiments using micro drones combined with multispectral sensors for remote sensing applications in the agricultural sector [13].

Sumnall et al. [14] estimated the Leaf Area Index (LAI) of a vineyard with a digital camera mounted on a micro-UAV. In addition, Primicerio et al. [5] mapped a vineyard through high-resolution multispectral images. Likewise, Pettorelli et al. [9] measured sunflower nitrogen status with microdrones and compared those measurements with data obtained from the ground-based platform. Remondino and Fraser [12] estimated the biomass and nitrogen content with a hyperspectral sensor mounted on a lightweight drone. Also, McCarthy and Halls [15] focused on the calculation of fluorescence light and temperature in order to predict the wateriest points in a field. They used data from hyperspectral sensor to calculate relationships between photosynthesis and chlorophyll fluorescence. Following the literature, we define the chosen indices to conduct the experiment.

### 2.1 Normalized Difference Vegetation Index (NDVI)

The Normalized Difference Vegetation Index (NDVI) is used to estimate the quantity, quality and development of vegetation based on different measures, through remote sensing from a space platform [16]. The NDVI measures the intensity of radiation in terms of several electromagnetic spectra bands which are constantly emitted or reflected by vegetation. In other words, plants absorb solar radiation in the photosynthetically radiation spectral region which they use as a source of energy in the process of photosynthesis [17]. Then the plant cells scatter that solar radiation in the infrared spectral region which is approximately half of the total solar energy. This is because the energy level per photon is not sufficient to synthesize organic molecules. The NDVI is calculated from these individual measurements as follows (1) [18]:

(1) $$\text{NDVI}\;=\;\left(\frac{\left(NIR-RED\right)}{\left(NIR+RED\right)}\right)$$

where RED and NIR stand for the different spectral reflectance measurements, and near-infrared regions, respectively. It is interesting to note that as a consequence, NDVI values range from -1.0 to +1.0 and can be calculated as long as the information linked to the near reflectance data and red is obtained [19]. In the presented research, this information is reflected in raster images in which different pixels present a reflectance value of the object captured by remote sensing. It should be mentioned that there are also other types of vegetation indices for different types of vegetation such as the Water Stress Index (WSI), the Soil Adjusted Vegetation Index (SAVI) or the Improved Vegetation Index (EVI) [20]. We must also consider that in this research the NDVI is presented following research conducted by Minařík and Langhammer [21] and Tucker [22].

### 2.2 Near-Infrared Spectroscopy (NIRS)

Near-Infrared Spectroscopy (NIRs) can be defined as an overtone absorption or combinations of vibrational bands that typically occur in the wavelength region of 780–3000 ηm [23].

In this sense, it should be remarked that the instruments for the NIR are similar to those used for UV-visible absorption spectroscopy. It should be pointed out that for this technique, the light sources are usually quartz cells or fused silica). These detectors used are usually lead sulfide photoconductors and some commercial spectrophotometers have already been designed to obtain NIR spectra [24]. This type of instrumentation is used for the quantitative determination of water in different samples, organic films and nitric acid to name a few. It should therefore be highlighted that near-infrared spectrophotometry is also often valuable for the determination and the optimization of primary or secondary amines in different mixtures, i.e. soil absorbance determination [25].

NIR has become a robust technique that permits to evaluate or reveal different properties related to moisture, oils or proteins which are used in agricultural products and in the fields in which they are executed as in the case of this research [26, 27, 28]). In order to know soil quality and functioning, a wide range of physical, chemical and biological properties must be analyzed. And as the possibilities of different properties are very high, it is interesting to use rapid and inexpensive techniques to characterize the soil and to be able to make applications in order to evaluate the impact of agriculture. In order predict maximum temperature reached on different soils, NIR technique has been widely used in recent decades [29].

### 2.3 Digital Elevation Model (DEM)

A Digital Elevation Model (DEM) is a numerical data structure which represents the spatial distribution of a quantitative variable [24]. DEM is a particular case because it is a variable represented by the terrain in relation to a specific reference system, but in the scientific literature there are different models for the same process: Digital Elevation Model (DEM), Digital Terrain Model (DTM) and Digital Surface Model (DSM).

Generally, the Digital Surface Model refers to the terrain surface and all objects on it, devoid of vegetation.

Regarding its application, it is a 3D digital computer representation of terrain surface adapted for use. Used in several fields and industries, it enables the extraction of parameters from agricultural land, topography layouts, water modelling or mass movement analysis - such as avalanches or earthquakes -creation of relief maps, flight planning, creation of physical models in order to recreate relief map models, mapping and flight simulations, and also precision agriculture, surface analysis and forest management mentioned in this study [1].

As a result of the application of the presented models in this research, three maps are utilized to assess the use of a vineyard; Vegetative state of crop, first, we analyze the map of the vegetative state of the crop in order to know its state, as well as its distribution. The results can determine the type of product that can be developed in different areas according to the analysis of the detected behaviors, as well as the identification of anomalous factors which may affect these crops [1, 4, 8, 9].

Second, Soil and plant moisture map. We decide to evaluation the soil and plant moisture map according to the information recorded by NIR. In this case, the aerial images of the vineyard, that is, the vine orchards, with the shape of the soil and the crop, are accompanied by contour lines of the soil. Therefore, it is possible to see and optimize the distribution of moisture in the plot and thus to analyze the evolution or optimization of surface runoff and micro topographies of the plot [3]. These soil and plant moisture maps are made to detect extensive plots with sprinkler irrigation systems that can help to detect damaged or blocked roads for the growing process [1]; Biomass density map, the presented study also analyzes the produced biomass density map in which the plot level can be observed through the completed flights. These flights can be performed at different heights to capture the canopy height, which results in the design of different biomass density maps that can be analyzed in order to optimize and improve the cultivation of the vineyard [1, 24].

Finally, shadows maps are also analyzed. These maps allow us to analyze the extent of the shadows produced by the lines and the crop height for each time of the year. By analyzing the distance from each of the streets to the height of the vineyard's orchard, it is possible to determine the right shade generation for the crop line and therefore to obtain lighter to optimize the cultivation process [1, 5].

### 2.4 A Cloud Computing software for multispectral images

Cloud computing is defined as a computing service from anywhere, using any mobile device through the Internet. It is provided through a type of parallel and distributed system on virtual interconnected computers which can be dynamically provisioned and presented as one or more unified computing resources based on Service Level Agreements (SLAs) established between the service provider and the user [18].

Compared to the traditional computing, Cloud Computing provides a systematic information which have a direct impact on the final recipient of the process in terms of effectiveness and analysis process. In this particular case, it helps the farmer monitor, almost instantaneously, the performance of his farm while providing decision support. The final recommendations will be given by an agronomist. For the presented study, we used the PIX4D software, specialized in aerial image processing.

Thanks to cloud computing technology, a farmer does not need to have all servers, hardware and software in the company's offices, as processes are carried out over the Internet [23]. All of this is on demand, and occurs in a dynamic and scalable way, to the extent that the needs for resource can increase or decrease according to the operation needs.

First, the data is captured from different remote devices, in this case a UAV was used remotely. Second, the collected data is automatically sent to the Cloud Computing servers then it is immediately processed with the power of the servers and Cloud processing. Third, the data processing is analyzed and optimized so that the results can be shared automatically.

The advantages of using the analysis power of a Cloud Computing software are: the farmer can see in a very intuitive way the collected data in this sample, compare with previous samples and quickly receive the first recommendations regarding irrigation, fertilizers, replanting, etc.

This process is performed under cloud virtualization technology. Through it, clients share a combination of servers, hard drives, communication devices, data devices and memory devices [6]. According to Niemeyer et al. [23] "Cloud computing has brought about new possibilities inbuilding and deploying computational infrastructures and complex services thanks to virtualization”.

These services are maintained and controlled by suppliers, but farmers only pay for what they use of these aforementioned computing resources and not according to the amount and duration of use. The cloud is the unique access point for the needs of all users where the only requirement is access to the Internet.

These features allow the farmer to access information quickly and anywhere on a smartphone or tablet, for example, the farm images and the necessary data to better management of it.

## 3 Methodology

Multispectral photogrammetry allows us to know the geometric properties of an object or a surface through the analysis of the information obtained by combining different images with high resolution information. That is, this technique can reconstruct different objects and surfaces to represent other factors of its structure to name a few [1].

Multispectral sensors can capture both visible and the invisible images of the spectrum. Thus, they provide data and calibrated information to monitor crops health [3]. These sensors can create panoramic RGB images of crops as well as support multispectral data from images taken from drones. This technology creates ortho mosaic images that enable measurements of an area. Multispectral imaging technology helps with land management, crop fertilization or early detection of biotic stress, to name a few. It can also help to monitor irrigation by identifying areas where water stress is suspected, to estimate crop yields and explore agronomic indices.

**Fig. 1:**
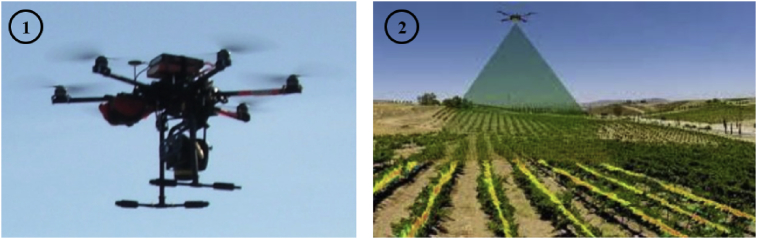
Surveying Operations: (1) UAV Hexacopter dji S900, (2) Scanning the terrain from UAV Hexacopter with the Parrot Sequioia system.

After the identification of the terrain and the planning of the flight, we collect raster information in order to generate fertilization maps, phytosanitary and hyperspectral analysis of the crop [1, 2, 3]. This method allows us to identify the causes of abnormal behavior and to know the exact cycle carried out in the crop in order to determine the optimal planting dates in each area of the plot or the correct amount of fertilizers, among others (See Fig. 1).

**Fig. 2:**
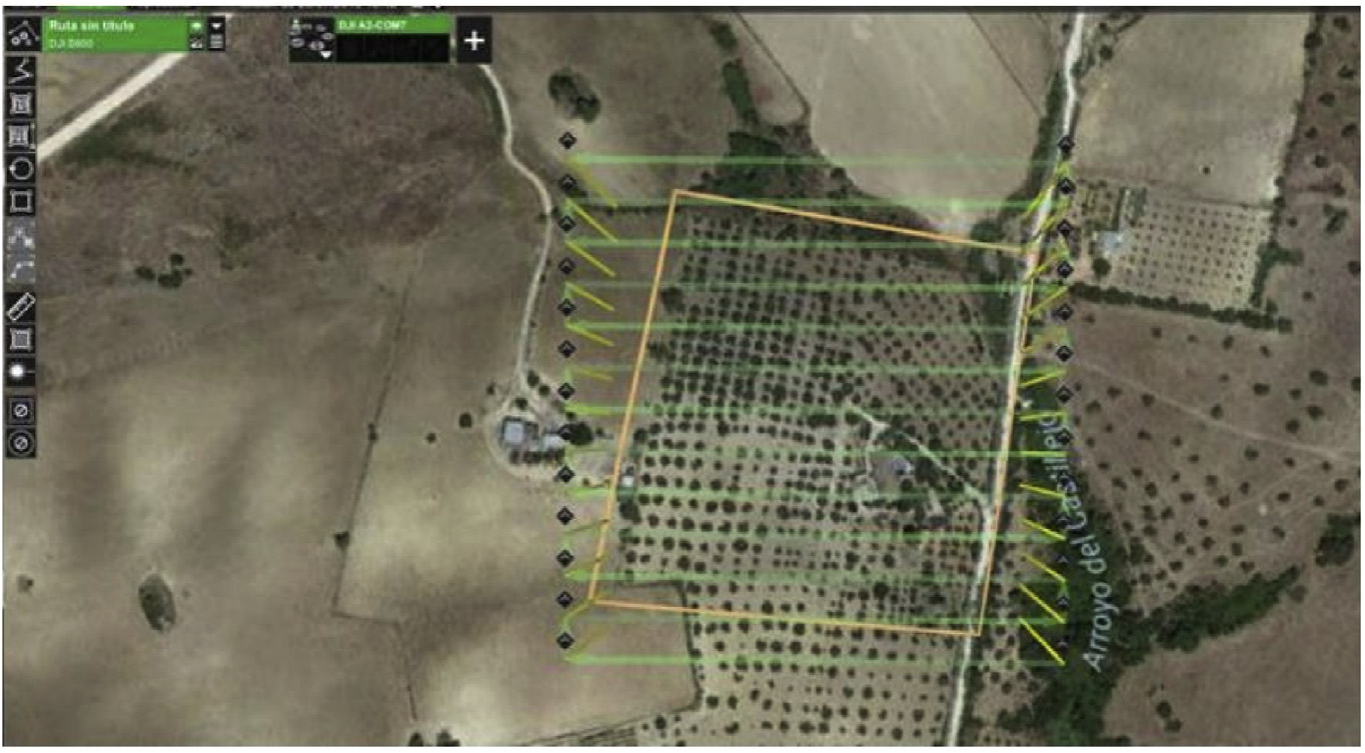
Satellite imagery of the studied area. Source: Google Maps [4]. Permission: ©2019 Google LLC, used with permission. Google and the Google logo are registered trademarks of Google LLC.

The analyzed crop area consists of 4 points. Theses colored waypoints are of 15 cm diameter each (See Fig. 2).

**Fig. 3:**
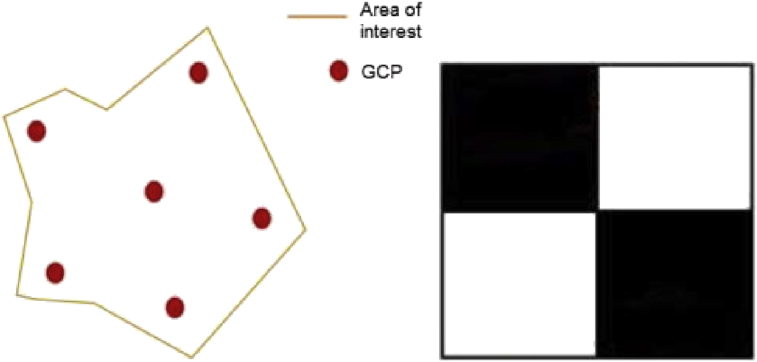
Waypoints distribution positioned on the vineyard ground. On the right: the waypoint visual GCP surveying equipment.

First, we placed some visual waypoints on the ground. These were used to ensure the accuracy of the geo-referencing of the product and were measured with a GPS surveying equipment and then positioned so that they covered all 4 corners of the vineyard area [2] (See Fig. 3).

**Fig. 4:**
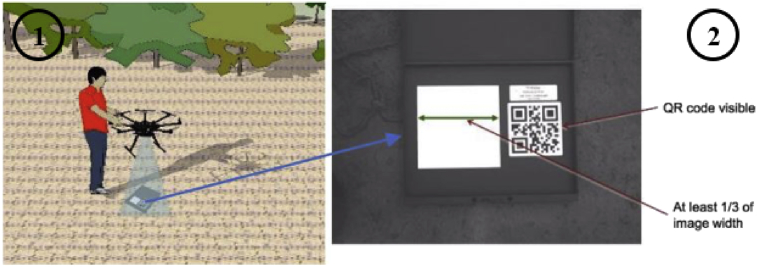
(1) Parrot Sequoia system, (2) Lighting panel system.

Then, the multispectral camera is calibrated in the field to adjust the sensors to the local lighting conditions. The on-board Parrot Sequoia sensor captures both RGB composition (visible light) and four separated bands: Green, Red, Red Edge and NIR wavelengths (See Fig. 4).

**Fig. 5:**
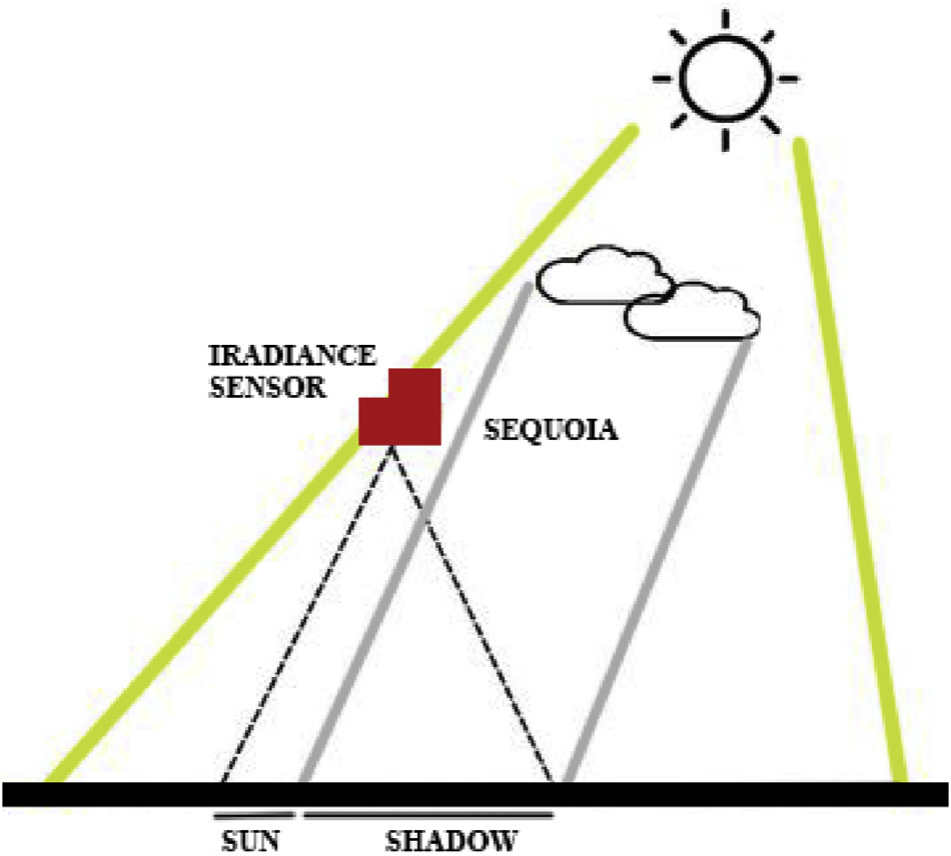
Lighting panel system.

The camera automatically captures the ratio of reflectance and corrects the recorded values to prevent it from cloud shadows (As shown in Fig. 5).

We introduce the flight planning in the internal computer of the UAV before the launch, the device itself controls the distribution of the images. It is essential to program them in the camera, depending on the overlaps and the desired precision. A limited overlap may imply that between two consecutive images and given the possible variations suffered by the UAV during the flight (considering two factors: the variations of the on-board GPS sensor or climatic conditions on the accuracy of the planned path), there is an uncovered area so that there are always planned overlaps both horizontally and vertically. when the route is made the UAV never leaves an uncovered space. The ground crew is always monitoring the device for safety reasons. Once the flights are over, we can download the aerial images of our onboard multispectral sensor.

### 3.1 Scenario of the survey: description of the study area

The study area is located in the northeastern part of Spain. The region is closed to the vineyards belonging to the D.O. Somontano, Tolva (Tolba in Catalan ribagorzano), a Spanish municipality in the province of Huesca (Aragon). The district extends along the middle valley of the Cajigar River which crosses the territory from north to south, between the eastern buttresses of the Sierra del Castillo de Laguarres (popularly known as Serra de l'Armellera) and the Ciscar Canyon. The Viacamp river and the San Cristóbal ravine flow to the left and the Riu Sec and l'Arguinoga to the right. Its relief is shaped, as its name suggests, like a hopper (funnel). The village is located in the centre of this hopper. The sampling area is approximately 1,169.85 m^2^.

**Fig. 6:**
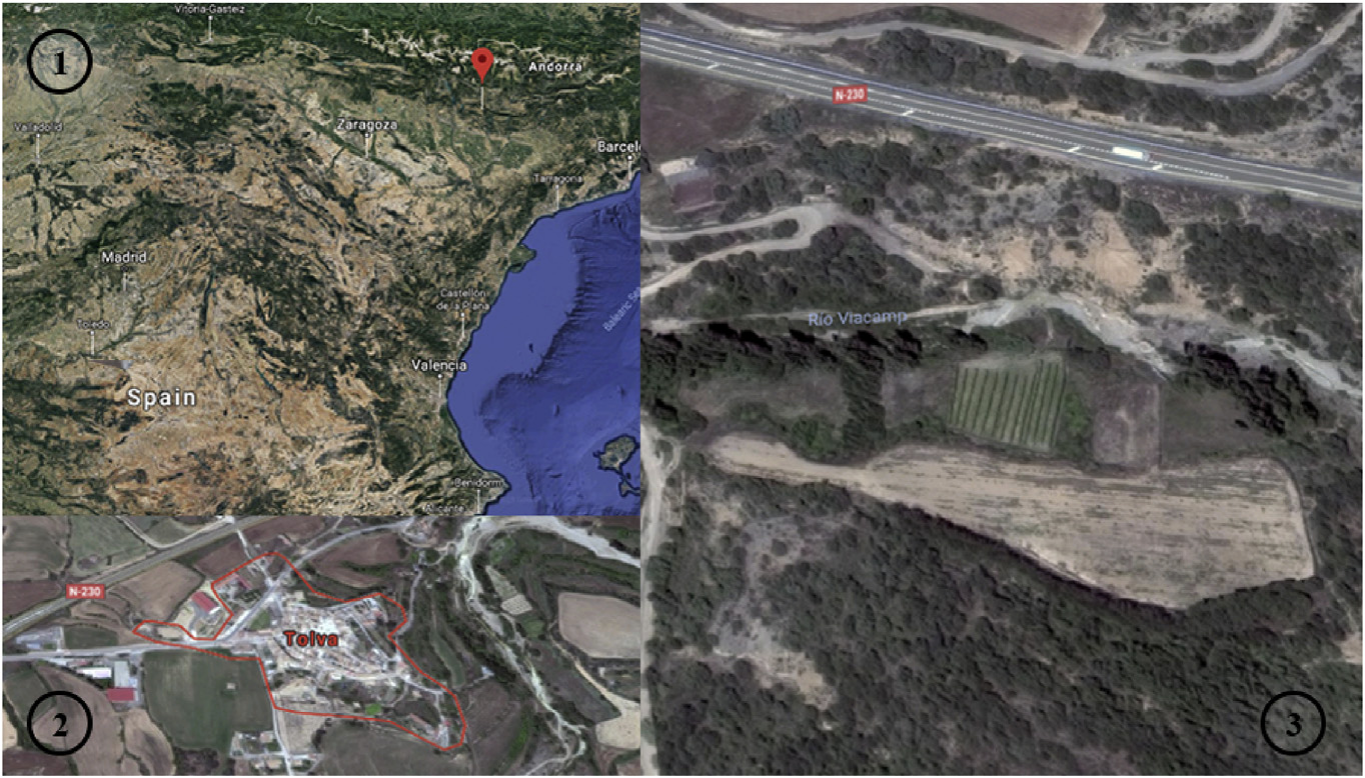
(1) Tolva location in Spain [31], (2) Tolva city in Aragon (Spain) [32], (3) Location map of the study area in Tolva [33]. Source: Google Maps [31–33]. Permission: ©2019 Google LLC, used with permission. Google and the Google logo are registered trademarks of Google LLC.

This terrain is used for wine production. It is a geographical area where earthquakes and landslides do not usually occur. It is a mixed area characterized by elevations and cliffs, which give way to small plains, slopes and valleys, where holm oaks, cereal fields and vineyards coexist. There is a river nearby but there is no possibility of flooding as the riverbed is scarce most of the year and can dry up almost completely in summer. The presented terrain is fed by an irrigation system and rainfall. The average number of daily sun hours is estimated to 6 to 8 per day and the average rainfall is 701 mm per year per rainfall. The coordinates of the landside area for this study are given as: 42.115556, 0.586086 (See Fig. 6).

### 3.2 Data collection

The data was collected during various flights conducted on August 10, 2017. The employed UAV was a hexacopter S900 model designed and produced by DJI and equipped with a multispectral camera installed underneath its platform. The hexacopter has a diameter of 120 cm and is equipped with a three-axis stabilized support (gimbal) to hold the camera. The flight duration is around 20–30 minutes depending on the weather conditions. The Parrot camera has a 16 MPIX RGB focal length and a pixel resolution of 4608 × 3456. Its fields of view are as follows: HFOV: 63.9°, VFOV: 50.1°, DFOV: 73.5°. It has also four 1.2 MPIX global shutter single band cameras: Definition: 1280 × 960 pixels, HFOV: 61.9°, VFOV: 48.5°, DFOV: 73.7°. The capture rate is 1 photo/s, which is stored in JPEG format [1, 2]. The remote flight control is performed through a DJI brand controller and the Mission Planner software. Although this UAV is capable of performing semi-autonomous, takeoffs and landings flights, it can be used remotely during flight.

In the presented study, the flight was performed in automatic mode, this means that once launched the UAV is capable of executing a pre-planned path since the GPS signal coverage allows us to conduct this type of flight with greater precision than if it were performed in an assisted or semi-assisted manner. The height of the flight is approximately 20 meters from the ground station with a spatial resolution of 4–5 cm per pixel [1].

### 3.3 The operator

**Fig. 7:**
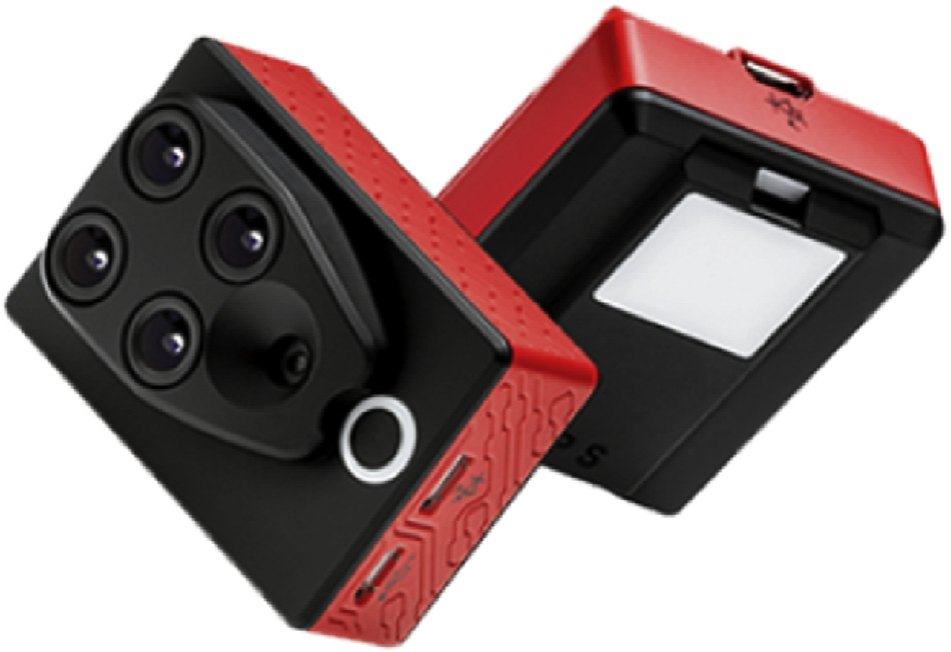
Multispectral Parrot Sequoia.

The used UAV is a DJI S900 hexacopter manufactured and developed by the DJI Brand, carrying a Parrot Sequoia multispectral camera (Fig. 7).

**Fig. 8:**
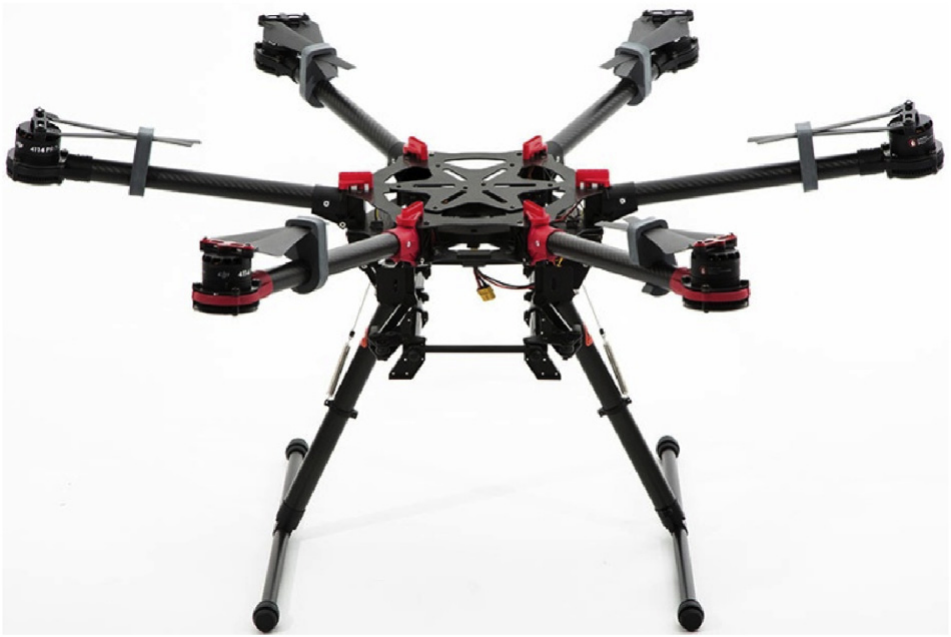
UAV hexacopter dji S900.

For the case study, the camera dimension and weight are: 75 mm × 39,6 mm × 18,5 mm (allowing a stable and quality flight when mounted) and 72 g. The camera has four 1.2 MP sensors (Green (550 BP 40), Red (660 BP 40), Red edge (735 BP 10), Near Infrared (790 BP 40) and a 16 MP RGB camera which allow us to capture 5 different images each time the lens shutter operates (As shown in Fig. 8). The camera, which records at the resolution indicated above both at slow and fast speeds, has its own stabilization system through its own IMU (Inertial Measurement Unit), which is an electronic device that measures and reports velocity, orientation and gravitational forces through the use of accelerometers and gyroscopes. Inertial measurement units are normally used to manage aircraft, including unmanned aerial vehicles (See Table 2).

**Table 2:** UAV hexacopter dji S900.

Drone	Manufacturer
Type	drone hexacopter
Dimension	Diameter 120cm, height 45 cm
Weight	9.4 kg with batteries (Maximum wight on fly 15 kg)
Engine power	6 electric brushless
Material	Carbon with aluminium alloy inserts
Payload	Ca. 500g.
Flight mode	Automatic with waypoint or based on radio control
Endurance	20–30 min (hovering flight time), 22 min
Ground Control Station	12-channels, 2.4 GHz modern, Telemetry for real time flight control and path tracking on video withing 2km
Flexible camera configurations	Digital gimbal with tri-axial roll and pitch control
Possible onboard imaging sensors	Photographic camera, video camera, thermal camera, multispectral camera

## 4 Analysis

### 4.1 Vegetative state of crop (NDVI)

As indicated before, the NDVI has been used in order to know the vegetation cover of the land. Apart from our experimental vines, there may be other herbaceous plants in the field, this may mean that we will have to advance or delay the cleaning and the land clearing depending on the obtained data, or on the other hand the experimental vines do not have the necessary nutrients. Thus, we can have two types of the vegetation cover which can vary from a leafier area to a drier area in the same crop, since in some cases the vegetation will be more compact and in others less dense and in areas with different plants.

Also, in case the seeds planted in the vineyard have evolved correctly, we can detect where they have been sown and have not grown. The NDVI is useful in detecting areas of high vegetation, or on the contrary those where vegetation is absent.

**Fig. 9:**
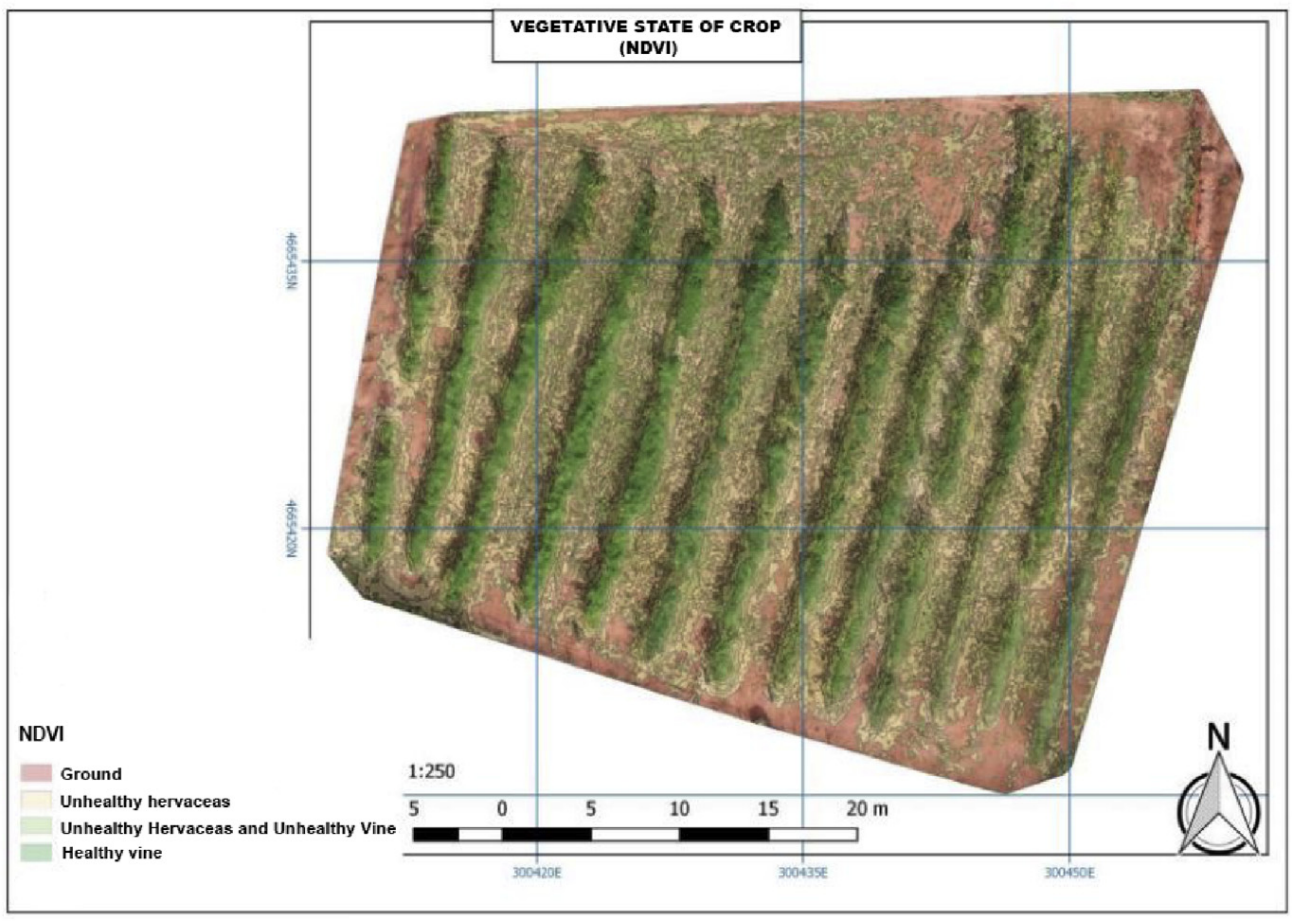
Vegetative state of crop (NDVI).

Regarding the vegetation cover coefficient, in Fig. 9 the red part of the figure corresponds to the experimental soil, and the green one corresponds to the vine.

In regard to the exploratory analysis, less vegetation appears in the more exposed area. The area of the plantation shows that the vineyard grows where there is less sunlight or levels of light. In this case and due to the characteristics of the plant, the vineyard grows under rain-fed, but the continuous exposure to sunlight does not benefit exclusively for it.

Fig. 9 indicates the samples of the vineyard lines, some parts are highlighted in red and some in green which show the current state of the vineyard. Below this level there are two green levels more, which correspond to unhealthy herbaceous and healthy herbaceous and unhealthy vine. If we place and overlap the two maps, 900 and the NDVI can be analyzed together.

### 4.2 Soil and plant moisture map (NIR)

**Fig. 10:**
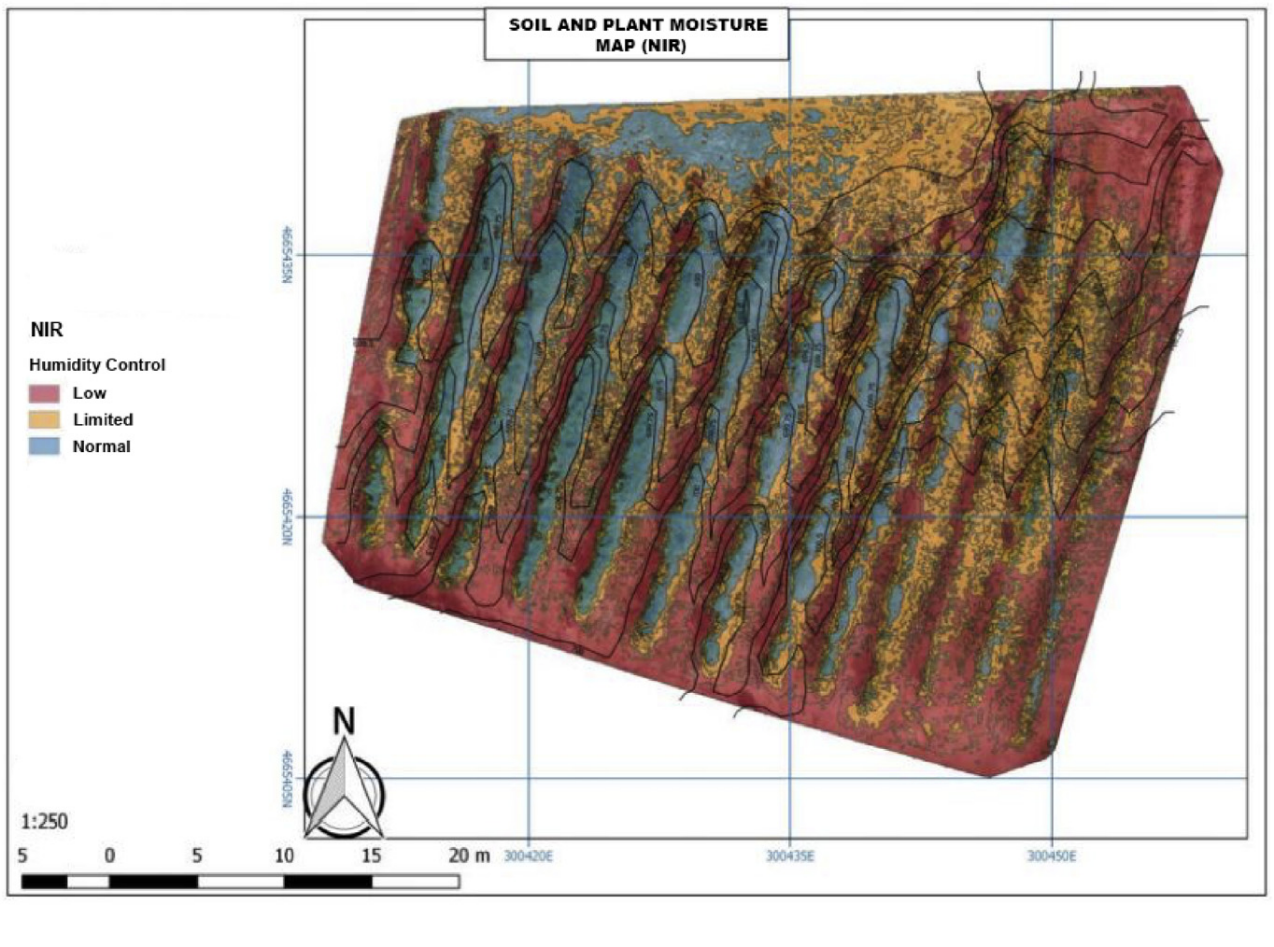
Soil and plant moisture map (NIR).

As mentioned before, soil and plant moisture map shows the amount of moisture in the plantation, as well as in the soil in which it is located. It should be pointed that the images are processed by NIR so that they can show each one of the indicators relative to soil and plant moisture. In order to generate the biomass map, moisture is detected from NIR images. Through the calculation of the NDWI (Normalized Water Differential Index) index, we can identify water masses and areas of high humidity saturation through the image analysis. In this way, we can use the index as a unit of measurement to determine the water stress in the vegetation, the saturation of humidity in the soil. Also, we can make direct delimitations of water masses such as lakes and reservoirs. This research is based on the combinations of bands NIR-Green, NIR-SWIR, SWIR-Green. All of these combinations of bands can be managed through the Geographic Information Systems (GIS) equipped with multispectral sensors. In this case, the ratio of multispectral bands is based on the NIR-Near Infrared (or near infrared) band and the SWIR- Short Wavelength InfraRed (short infrared) band. The equation is based on the relationship between the differences reflected in each of the resulting infrared colors in Fig. 10. This map has been generated with this technique using PIX4D software, specialized in aerial image processing (2):

(2) $$\text{NDWI}\;=\;\left(\frac{\left(NIR-SWIR\right)}{\left(NIR+SWIR\right)}\right)$$

Therefore, Fig. 10 shows the driest areas of the vineyard or those which have less moisture and are close to the crop. Also, we can see that, if they were irrigated crops, there would be very extent areas with less moisture. Thus, we would have areas with less moisture or even water for the correct growth of the plantation, due to the characteristics of the soil, for example, in matters related to the drainage of the soil.

From Fig. 10, the study area needs to be irrigated more in order to identify the area that need the most water and where the moisture must be increased accordingly. In addition, this Figure indicates the moisture degree of the vineyard in terms of low, scarce or standardized levels.

The red color represents its moisture which coincides with the sunny area of the plantation, that is to say, the moisture values are low where the light strikes more. Also, the orange corresponds to the middle area with low moisture values that also coincides in terms of light between medium and low. It is important to note that the blue part represented in Fig. 10 is where the highest moisture is found and where are furrows between the vines.

This exploratory analysis makes it possible to identify within which areas need to increase moisture in order to optimize planting, therefore, increase crop productivity.

### 4.3 Biomass density maps (MDE-MDT)

With the analysis of the produced biomass density, references can be based on cubic meters levels to determine the produced biomass by each area of the vineyard. For the development of this map, the place where solar incidence sensors are established must be optimized and executed after a deep measurement process in order to avoid fake measurements. Biomass sensors capture how the sun hits each of the measuring points and sensors to avoid wrong biomass data. In addition, when mapping the produced biomass, it is possible to observe the indicators collected at different times of the day, according to the sunlight reflectance on the experimental vineyard. This process aims to determine biomass levels produced at different times of the day and according to the type of light that strikes each of the terrain. To develop this map, we have also followed Gao's technique [30] and PIX4D software has been used.

**Fig. 11:**
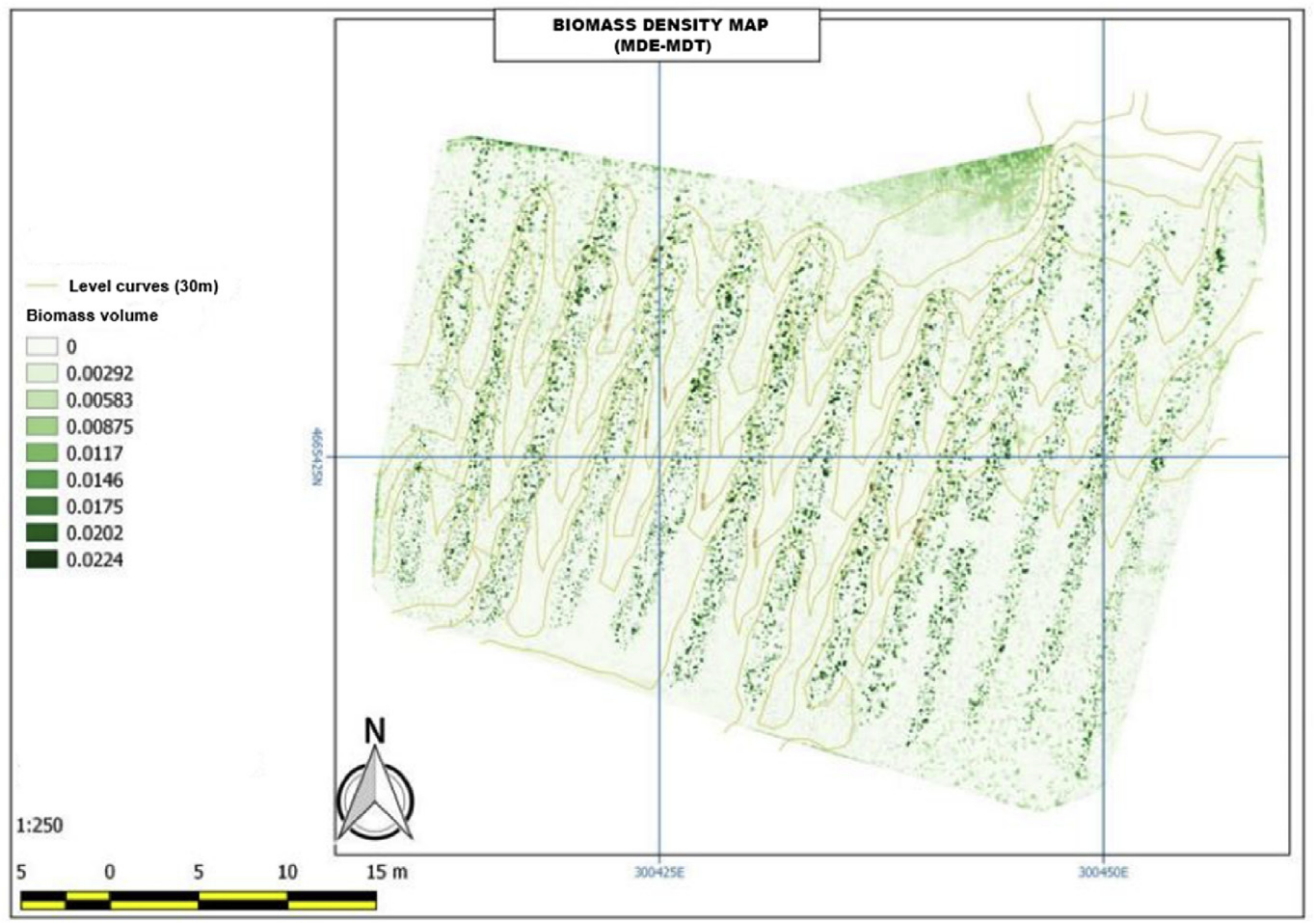
Biomass density map (MDE-MDT).

In this case, the biomass map indicates the green color range the percentage of generated biomass by each of the lines of vineyards within the green color range. From this analysis we can determine the vineyards which are producing the most biomass and, therefore, developing each of the objectives related to the production. The results of this study help vineyard owners to improve and monitor every area of their yield. Fig. 11 shows the experimental site according to the biomass level.

### 4.4 Shadows maps

As mentioned before, the shadow map presents the information provided by the produced biomass and the vegetative state of the crop. It reveals the health of the vegetation with respect to the production objectives set by the owners.

Concerning shadow map, the vegetative state of the crop helps to meet the requirements of those nutrients that the crop needs. For the calculation, it is necessary to know the needs of the crop since, at each phenological development stages of the species, these have different singularities such as the sowing time in different seasons or the pathogens previously treated. In addition, the number of fertilizers and the time taken for their release are also considered.

**Fig. 12:**
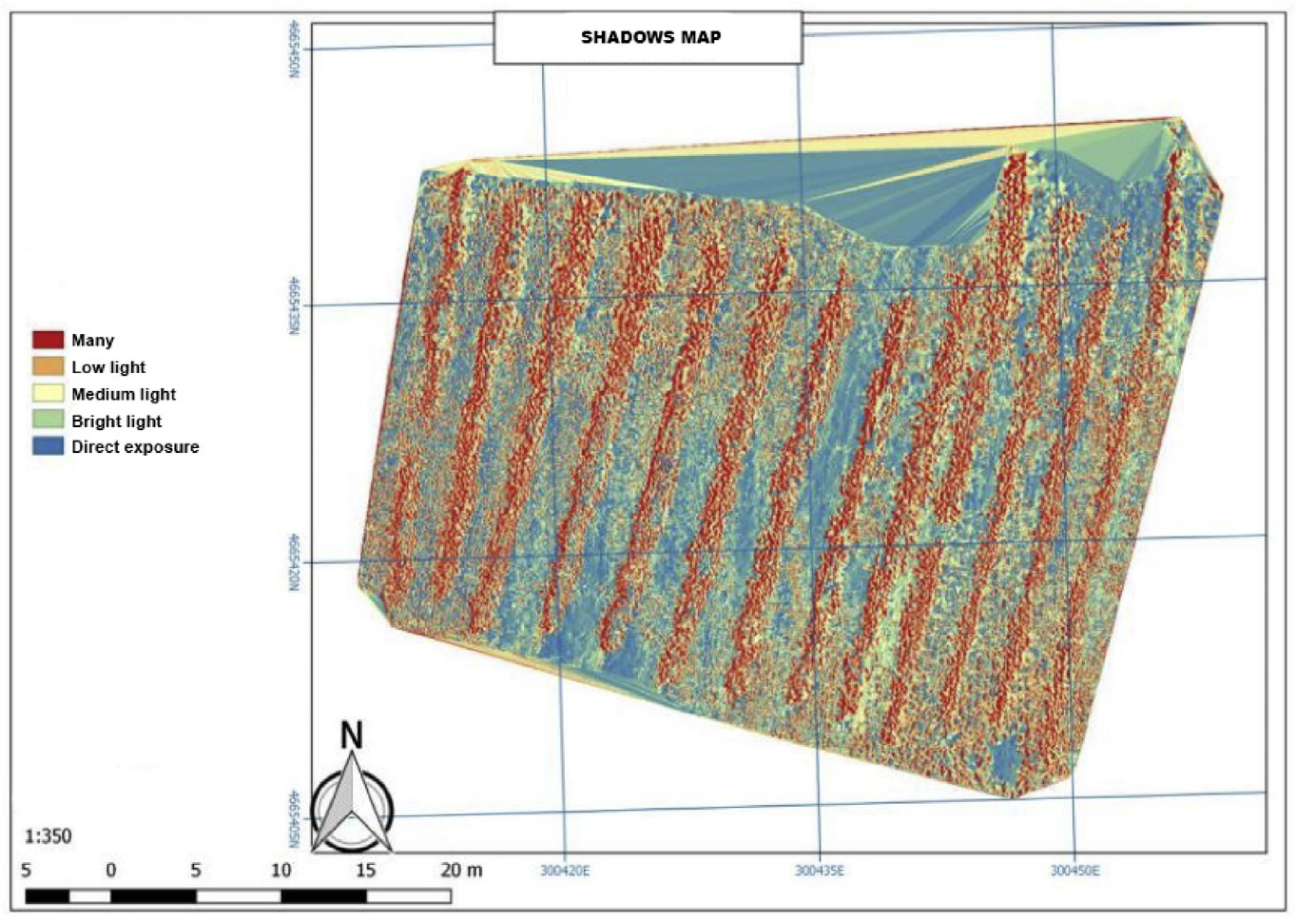
Shadows map.

Through the analysis of the biomass, the shadow map calculates the measurement of the raster of the generated cartographies and the obtained information enables to exploit the fertilizer maps for each of the plots. In this sense, Fig. 12 shows how the solar radiation hits the ground.

As we already know, agriculture and crops are affected by the sunlight as well as the soil and the plant. Fig. 12 shows a prominent area in blue totally exposed to the sunlight over the time study. On the other hand, in the red area, it should be noticed that the sunlight does not hit it, that is, there is shadow. As a result, there will be less solar irradiance and plant growth will be lower. It is important to analyze this fact because his type of maps help to identify how they affect the global level in terms of land sowing (see Fig. 12).

## 5 Discussion & conclusion

As seen throughout the research, this study aims to analyze through mounted remote sensing drones the different issues related to the development of vineyard in order optimize and increase their production. It also analyzes the vegetative state map of the crop, as well as soil and plant moisture map, the biomass volume and shadow maps. The experiment develops the technologies already presented to show the results of the research and which are specifically NDVI, NIRS and DEM. To conduct the study, the experimental site is selected and the drone, in which a remote sensing camera is installed, is prepared.

The union of the information provided by the generated biomass and the vegetative state of the plant allows us to develop the idea of possible needs and nutrients for the crop. In this sense and given the results of the research to calculate the needs of the crop, the phenological development of the species and its singularities should be considered. The analysis of the 4 maps presents those areas more exposed to sunlight, in which there is less moisture, either by evaporation or drainage, and in which there is less vegetation.

At the same time, there is a solar irradiance related to moisture. With the exploratory analysis of the results, we can glimpse the vegetation cover in which herbaceous and unhealthy crops will be seen. Likewise, it has been possible to detect which is the space in which there is less solar incidence, mainly due to the fact that the crop generates its own shade and is cooled providing more humidity and giving rise to the healthiest cultivation of the vines. In this case, and as a result of the research, it has been observed that there is shade in those places where humidity increases. As a result of the experiment it has also been possible to observe the increase the shadow, and its link with the percentage of light that hits the ground.

A priori, after the results of the experiment, we may think that the vineyard is dry. However, when we carry out the proposed experiments and make an exhaustive analysis, we can see that there is more moisture than human eye can see. Therefore, the identified micro contexts which help to generate heatmaps, enable us to can carry out an analysis of the real state of the vineyard as a whole.

We can also conclude that all the maps should be developed together (moisture map, biomass density mail, soil and plant moisture map, the vegetation status of the crop) in this type of analysis area, in order to have a complete state of the soil under study.

The results of this experiment can be used by professionals who want to improve their self-consumption or those who want to produce optimal vineyards. They can also use these results to improve their crops, use UAV and remote sensing technology to optimize and increase crop yields. The results of the study reveal an exploratory analysis of the vegetative state of a vineyard, which can increase and enrich the literature in this field. The limitations of the research are those directly linked to the explored terrain and the climatic conditions of the country in which the analysis was conducted.

## Declarations

### Author contribution statement

Jose R. Saura: Conceived and designed the experiments; Performed the experiments; Analyzed and interpreted the data; Wrote the paper.

Ana Reyes-Menendez: Conceived and designed the experiments; Performed the experiments; Analyzed and interpreted the data; Contributed reagents, materials, analysis tools or data.

Pedro Palos-Sanchez: Conceived and designed the experiments; Performed the experiments; Analyzed and interpreted the data.

### Funding statement

This research did not receive any specific grant from funding agencies in the public, commercial, or not-for-profit sectors.

### Competing interest statement

The authors declare no conflict of interest.

### Additional information

No additional information is available for this paper.

## References

[1] Candiago S., Remondino F., Giglio M.D., Dubbini M., Gattelli M. (2015). Evaluating multispectral images and vegetation indices for precision farming applications from UAV images. Rem. Sens..

[2] Santhosh K.S., Soizik L., Grant M.C., George A.S. (2003). Remote sensing applications for precision agriculture: a learning community approach. Remote Sens. Environ..

[3] David J.M. (2013). Twenty-five years of remote sensing in precision agriculture: key advances and remaining knowledge gaps. Biosyst. Eng..

[4] Google Maps (2019). Satellite Imagery of the Studied Area. https://goo.gl/maps/qXhKW82jgJy.

[5] Primicerio J., Di Gennaro S.F., Fiorillo E., Genesio L., Lugato E., Matese A., Vaccari F.P. (2012). A flexible unmanned aerial vehicle for precision agriculture. Precis. Agric..

[6] Reyes-Menendez A., Saura J., Alvarez-Alonso C. (2018). Understanding world environment day user opinions in twitter: a topic-based sentiment analysis approach. Int. J. Environ. Res. Public Health.

[7] Grenzdörffer G.J., Engel A., Teichert B. (2008). The photogrammetric potential of low-cost UAVs in forestry and agriculture. Int. Arch. Photogramm. Remote Sens. Spat. Inf. Sci..

[8] Ouédraogo M.M., Degré A., Debouche C., Lisein J. (2014). The evaluation of unmanned aerial system-based photogrammetry and terrestrial laser scanning to generate DEMs of agricultural watersheds. Geomorphology.

[9] Pettorelli N., Vik J.O., Mysterud A., Gaillard J.M., Tucker C.J., Stenseth N.C. (2005). Using the satellite-derived NDVI to assess ecological responses to environmental change. Trends Ecol. Evol..

[10] Gitelson A.A., Kaufman Y.J., Merzlyak M.N. (1996). Use of a green channel in remote sensing of global vegetation from EOS-MODIS. Remote Sens. Environ..

[11] Turner D., Lucieer A., Malenovsky Z., King D.H., Robinson S.A. (2014). Spatial co-registration of ultra-high resolution visible, multispectral and thermal images acquired with a micro-UAV over Antarctic moss beds. Rem. Sens..

[12] Remondino F., Fraser C. (2006). Digital camera calibration methods: considerations and comparisons. Int. Arch. Photogramm. Remote Sens. Spat. Inf. Sci..

[13] Remondino F., Spera M.G., Nocerino E., Menna F., Nex F. (2014). State of the art in high density image matching. Photogrammetria.

[14] Sumnall M.J., Fox T.R., Wynne R.H., Blinn C., Thomas V.A. (2015). Estimating leaf area index at multiple heights within the understorey component of Loblolly pine forests from airborne discrete-return lidar. Int. J. Remote Sens..

[15] McCarthy M.J., Halls J.N. (2014). Habitat mapping and change assessment of coastal environments: an examination of worldview-2, quickbird, and ikonos satellite imagery and airborne lidar for mapping barrier island habitats. ISPRS Int. J. Geo-Inf..

[16] Palos-Sanchez P., Saura J.R., Reyes-Menendez A., Esquivel I.V. (2018). Users acceptance of location-based marketing apps in tourism sector: an exploratory analysis. J. Spatial Organ. Dynam..

[17] Klemas V. (2013). Remote sensing of emergent and submerged wetlands: an overview, international journal of remote sensing. Int. J. Remote Sens..

[18] Paloz-Sanchez P.R., Arenas-Marquez F.J., Aguayo-Camacho M. (2017). Cloud computing (SaaS) adoption as a strategic technology: results of an empirical study. Mobile Inf. Syst..

[19] Minařík R., Langhammer J. (2016). Use of a multispectral UAV photogrammetry for detection and tracking of forest disturbance dynamics. ISPRS - International Archives of the Photogrammetry, Remote Sensing and Spatial Information Sciences, XLI-B8.

[20] Tucker C. (1979). Red and photographic infrared linear combinations for monitoring vegetation. Rem. Sens. Environ..

[21] Weinmann M., Weinmann M. (2017). Geospatial computer vision based on multi-modal data—how valuable is shape information for the extraction of semantic information?. Rem. Sens..

[22] Bareth G., Aasen H., Bendig J., Gnyp M.L., Bolten A., Jung A., Michels R., Soukkamäki J. (2015). Low-weight and UAV-based hyperspectral full-frame cameras for monitoring crops: spectral comparison with portable spectroradiometer measurements. PFG Photogramm. Fernerkund. Geoinf..

[23] Niemeyer J., Rottensteiner F., Soergel U., Heipke C. (2015). Contextual classification of point clouds using a two-stage CRF. Int. Arch. Photogramm. Remote Sens. Spat. Inf. Sci..

[24] Guignard S., Landrieu L. (2017). Weakly supervised segmentation-aided classification of urban scenes from 3D LiDAR point clouds. Int. Arch. Photogramm. Remote Sens. Spat. Inf. Sci..

[25] Monnier F., Vallet B., Soheilian B. (2012). Trees detection from laser point clouds acquired in dense urban areas by a mobile mapping system. Int. Arch. Photogramm. Remote Sens. Spat. Inf. Sci..

[26] Zou X., Zhao G., Li J., Yang Y., Fang Y. (2016). 3D land cover classification based on multispectral LiDAR point clouds. Int. Arch. Photogramm. Remote Sens. Spat. Inf. Sci..

[27] Ahokas E., Hyyppä J., Yu X., Liang X., Matikainen L., Karila K., Litkey P., Kukko A., Jaakkola A., Kaartinen H. (2016). Towards automatic single-sensor mapping by multispectral airborne laser scanning. Int. Arch. Photogramm. Remote Sens. Spat. Inf. Sci..

[28] Matikainen L., Hyyppä J., Litkey P. (2016). Multispectral airborne laser scanning for automated map updating. Int. Arch. Photogramm. Remote Sens. Spat. Inf. Sci..

[29] Matikainen L., Karila K., Hyyppä J., Litkey P., Puttonen E., Ahokas E. (2017). Object-based analysis of multispectral airborne laser scanner data for land cover classification and map updating. ISPRS J. Photogrammetry Remote Sens..

[30] Gao B. (1996). NDWI - a normalized difference water index for remote sensing of vegetation liquid water from space. Rem. Sens. Environ..

[31] Google Maps (2019). Satellite imagery of the studied area. https://goo.gl/cGh5kD.

[32] Google Maps (2019). Satellite imagery of the studied area. https://goo.gl/maps/zFzMndwtYMJ2.

[33] Google Maps (2019). Satellite imagery of the studied area. https://goo.gl/maps/qXhKW82jgJy.

